# Epigallocatechin-Gallate (EGCG): An Essential Molecule for Human Health and Well-Being

**DOI:** 10.3390/ijms26189253

**Published:** 2025-09-22

**Authors:** Emanuele Rovaldi, Violante Di Donato, Giovanni Paolino, Marzia Bruno, Alessia Medei, Steven Paul Nisticò, Giovanni Pellacani, Norbert Kiss, Giulia Azzella, Andras Banvolgyi, Carmen Cantisani

**Affiliations:** 1 Dermatology Unit, Department of Medical and Cardiovascular Sciences, “Sapienza” University of Rome, 00161 Rome, Italy; emanuele.rovaldi@gmail.com (E.R.); pamaca@tiscali.it (M.B.); steven.nistico@uniroma1.it (S.P.N.); giovanni.pellacani@uniroma1.it (G.P.); azzellagiulia1996@gmail.com (G.A.); 2 Department of Obstetrics and Gynecology, University Sapienza of Roma, 00185 Rome, Italy; violante.didonato@uniroma1.it; 3 Unit of Dermatology, IRCCS, Ospedale San Raffaele, 20132 Milano, Italy; paolino.giovanni@hsr.it; 4 Department of Radiology, Ospedale Santa Maria, University Sapienza of Perugia, 05100 Terni, Italy; alessiamedei94@gmail.com; 5 Department of Dermatology, Venereology and Dermatooncology, Semmelweis University, 1085 Budapest, Hungary; norbert.f.kiss@gmail.com (N.K.); banvolgyi.andras@med.semmelweis-univ.hu (A.B.)

**Keywords:** aging, cancer, catechin, flavonoid, green tea, polyphenols

## Abstract

Green tea, long consumed across Southeast Asia, is highly esteemed for its medicinal properties and is often favored over conventional treatments in Eastern cultures. Its health benefits are largely attributed to its minimal processing, which preserves pharmacologically active compounds, particularly catechins, a key class of polyphenols, with epigallocatechin-gallate (EGCG) being the most abundant and bioactive. These compounds exhibit antioxidant, anti-cancer, antimicrobial, and antiangiogenic properties. Beyond systemic health, EGCG has diverse applications in dermatology, including the treatment of viral warts, psoriasis, lichen sclerosus, acne, vaginal dryness, alopecia, and UV-induced skin damage. Emerging research also highlights its promise in aesthetic medicine for mitigating skin oxidative stress, improving skin brightness and neutralizing free radicals, responsible for wrinkles, hyperpigmentation, and loss of elasticity. The aim of this review is to examine the most recent literature on the wide-ranging clinical applications of Epigallocatechin-gallate (EGCG) and to assess its potential use as a daily foundational supplement to enhance both physical and mental well-being, focusing on the dermatological benefits.

## 1. Introduction

**Figure 1:**
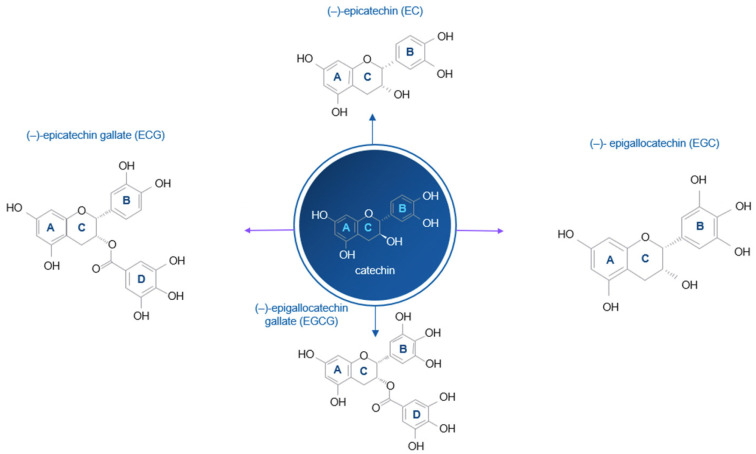
Chemical structure of the main catechins contained in green tea: the common chemical structure of a catechin molecule consists of two benzene rings (A- and B-rings) and a dihydropyran heterocyclic ring (C-ring) with a hydroxyl group at carbon 3. Epicatechin gallate (ECG), containing Gallate group and hydrogen group; Epicatechin (EC), containing two hydrogen groups; Epigallocatechin (EGC), containing one hydrogen group and one hydroxyl group; Epigallocatechin gallate (EGCG), containing Gallate group and hydroxyl group.

According to a Chinese legend, tea was discovered around 4000 years ago by Emperor Shen Nung. One day while he was boiling water, leaves from a nearby tea tree were blown into his pot by the wind. Intrigued by the aroma and flavor, he tasted it, leading to the accidental discovery of tea. Tea, derived from “*Camellia sinensis*” (family *Theaceae*), is one of the most consumed beverages. The three main types of tea are green, oolong and black tea. Green tea is made by quickly processing fresh leaves to avoid fermentation. Oolong tea is partially fermented, while black tea is fully fermented before drying. Black tea is the most consumed (78%), in particular in the U.S., Europe, Africa, and India. Green tea accounts for 20% of consumption, mainly in China and Japan, and oolong tea, mostly from Southern China (2%). Green tea comes in many varieties, each shaped by the culture, climate, and processing techniques of the region in which it is grown. The two most famous producers are China and Japan, though other countries like Korea, Vietnam, India, and Sri Lanka also contribute unique green teas to the global market. In Japan, green tea is steamed rather than pan-fired, giving it a characteristic grassy, vegetal, and umami-rich flavor. The most common type is Sencha, which is grown throughout regions like Shizuoka, Uji (Kyoto), and Kagoshima. When tea leaves are grown in the shade for several weeks before harvest, the result is Gyokuro, a luxurious tea with a deep, sweet, and savory taste. The same shaded leaves can be ground into a fine powder to make Matcha, which is used in traditional tea ceremonies as well as modern lattes and desserts. The unground leaf used for matcha is called Tencha. Other notable Japanese green teas include Bancha, made from later harvests and offering a more robust, earthy flavor; Kukicha, which is composed of twigs and stems; and Genmaicha, a blend of green tea with roasted brown rice, producing a nutty and toasty profile. A unique style is Hojicha, which involves roasting the tea leaves to create a warm, caramel-like aroma and flavor. China, the birthplace of tea, has a wide range of green teas, often pan-fired, which impart nutty, roasted, or chestnut-like flavors. Among the most celebrated is Longjing, also known as Dragon Well tea, grown near Hangzhou in Zhejiang province. It is known for its flat leaves and rich, mellow flavor. Tea cultivation in Europe is a relatively new but steadily growing phenomenon. While Europe has long been a continent of tea drinkers, especially in countries like the UK and Russia, the actual farming of tea has traditionally been limited due to climatic constraints. However, in recent decades, a number of regions across Europe have started experimenting with growing *Camellia sinensis*, thanks to the warming effects of climate change, increased agricultural experimentation and a rising interest in sustainable local food production. The northern regions of Italy, especially around Lake Maggiore in Piedmont and Lombardy, have become key areas for tea experimentation. These zones benefit from mild, humid microclimates, with rich, acidic soils and relatively stable rainfall patterns, all favorable conditions for tea cultivation. In Tuscany, known for its fertile land and expertise in high-value crops like wine and olive oil, small plots of tea are being cultivated using biodynamic and organic methods. Even in southern regions like Sicily, where the climate is generally warmer and drier, tea is being grown successfully in mountainous or coastal zones that offer cooler temperatures and higher humidity. Usually, tea is prepared using tea bags steeped in hot water at a ratio of 1 g of leaves per 100 milliliters of water, yielding a 0.35% solid concentration. Green tea infusions contain a good amount of catechins (about 30%) and flavonols (2%) (antioxidant molecules with unpaired electrons, which neutralize free radicals by donating an electron or a hydrogen atom from their molecular structure), while black tea contains less catechins (around 9%), but includes theaflavins and flavonols (3%). Green tea is particularly beneficial thanks to its minimal processing, which preserves its pharmacologically active compounds. Polyphenols, especially catechins, make up around 30% of green tea’s content. Key catechins include epicatechin (EC), epicatechin gallate (ECG), epigallocatechin (EGC), and epigallocatechin gallate (EGCG). On the other hand, black tea, in comparison, has about 9% catechins. EGCG is the most present (10–15%), followed by EGC (6–10%), ECG (2–3%), and EC (2%) [1,2]. Catechins, including EGCG (epigallocatechin gallate), share a common chemical structure that defines their biological activity, particularly their role as antioxidants. Chemically, catechins are part of the flavonoid family, more specifically the flavan-3-ols subgroup. Their structure is based on a three-ring system: two aromatic rings and one heterocyclic ring. This core structure is further modified by the presence of multiple hydroxyl (–OH) groups, which are crucial to their chemical reactivity and ability to neutralize free radicals (Figure 1). As antioxidants, they donate electrons or hydrogen atoms to free radicals. By doing so, they stabilize the radicals, preventing them from attacking important cellular components like DNA, proteins, and lipids. This protective mechanism helps preserve cellular integrity and reduces the risk of mutations or inflammation triggered by oxidative damage. In particular, EGCG is considered the most active catechin in green tea due to its unique chemical structure, high concentration and broad biological activity. One of the main reasons that EGCG is more active is its chemical structure, which includes a gallate group (GA) attached to carbon 3′ of the C-ring [3]. This gallate group, along with trihydroxyl (–OH) structure on the aromatic B-ring (carbons 3′, 4′, and 5′) and the presence of the gallate ester on the C-ring, gives EGCG a higher capacity to donate electrons [4]. That means it can neutralize a wider range of free radicals more effectively than other catechins. It also acts as a chemo-preventive, anti-cancer, antimicrobial, antiangiogenetic agent [5,6,7]. These compounds are water-soluble, colorless, and contribute to the bitterness and astringency of tea. Other minor but notable components include flavonols like quercetin (an antioxidant which inhibits enzymes like cyclooxygenase (COX) and lipoxygenase (LOX), theobromine, theophylline (two mild stimulants that block adenosine receptors in the brain and other tissues) and phenolic acids, like gallic acid (characterized by a phenol ring, an aromatic ring with at least one hydroxyl group (–OH), attached to a carboxylic acid group (–COOH)) [8].

## 2. Methods of EGCG Intake

A typical cup (250 mL) of green tea contains around 50–100 mg of EGCG and 30–40 mg of caffeine. According to the European Food Safety Authority (EFSA), the average daily intake of EGCG in adults from tea is estimated at 90–300 mg (about 4 cups a day). However, consuming 800 mg or more per day may increase liver enzyme levels, which could be a sign of liver stress. To maximize the health benefits of epigallocatechin gallate (EGCG), it’s important to consider the most effective methods of intake, as its absorption and stability can be influenced by several factors. Drinking green tea is the most natural and common way to consume EGCG. However, the concentration of EGCG can vary depending on the type of tea, brewing time and water temperature, but the dose is often not sufficient. For optimal results, you should use high-quality green tea leaves or matcha powder, brew with water between 70 and 80 °C (158–176 °F) to preserve catechins, steep for 2–3 min to extract beneficial compounds without making the tea too bitter. Tea should be consumed while fasting or, at the very least, away from meals and taking a “tea-break” [9]. On an empty stomach, EGCG is best absorbed, as food, especially proteins and dairy, can reduce its bioavailability. However, taking it this way may cause stomach discomfort or liver stress in sensitive individuals or at high doses, so it is best to start with lower amounts and monitor your body’s response [10]. Catechins—especially EGCG—are commonly used in food manufacturing, including products like instant powders and baked goods such as bread [11]. However, catechins are prone to epimerization and can degrade significantly during processing, leading to substantial loss of their content [12]. Their stability in food is affected by several factors, including temperature, pH levels, the presence of metal ions, and exposure to oxygen. However, taking it this way may cause stomach discomfort or liver stress in sensitive individuals or at high doses, so it is best to start with lower amounts and monitor your body’s response.

EGCG is also available in capsule or tablet form, often standardized for consistent dosing. This allows for higher and more controlled intake, especially for therapeutic purposes. Many times, they are enteric-coated capsules combined with Vitamin C or quercetin to enhance absorption. For skin-related benefits (e.g., anti-aging, anti-inflammatory, or depigmenting effects), EGCG can also be applied directly to the skin through serums or creams. Topical formulations often use stabilized or glucosylated EGCG (like EGCG-G1) to enhance skin penetration and efficacy [13,14,15].

## 3. Biophysical Properties for Pharmaceutical Uses

Temperature plays a key role in how EGCG undergoes both oxidation and epimerization. When heated to 80 °C, EGCG levels steadily declined over time across all pH levels tested. Interestingly, the concentration of its epimer, gallocatechin gallate (GCG), initially rose to a peak, but then also declined as heating continued. Meanwhile, levels of gallic acid (GA), a degradation product, increased steadily, indicating breakdown of the catechins. Overall, the combined amount of EGCG and GCG consistently dropped during the heating period. These findings align with earlier studies that observed similar temperature effects on catechin degradation and epimerization across both moderate and high temperature ranges (25–165 °C) [10]. Despite prolonged heating, there was no statistically significant change (*p* > 0.05) in the pH of the EGCG buffer solutions, suggesting that the buffering capacity remained stable. The breakdown of EGCG and GA was shown to be pH-dependent, with faster degradation at higher pH levels. In contrast, at acidic pH levels below 4, EGCG remained more stable, with minimal changes in the concentrations of EGCG, ECG, and GA. In a solution of pH 1.6, EGCG was stable for up to 96 h and stability decreased with increasing pH of the solution. In fact, no notable differences were seen between pH 2.2 and 3.0, supporting findings by Li et al. (2012) [16]. These results highlight the importance of both temperature and pH in preserving the stability of EGCG in food processing and storage. Since EGCG has well-documented health benefits, maintaining its integrity is essential, especially in products like tea and supplements. The data suggest that acidic environments and lower temperatures are most effective in slowing down EGCG degradation and preserving its antioxidant potential. This has practical implications, because it makes it very difficult to use EGCG in sunscreen or lotions, where antioxidant and anti-inflammatory effects might be very useful. Due to its strong hydrating properties, in the future, hyaluronic acid could improve the antioxidant effects of the EGCG for a higher skin permeation [17].

## 4. Pharmacokinetic Profile of EGCG

Recent studies have investigated the pharmacokinetic profile of orally administered EGCG in healthy individuals. EGCG is rapidly absorbed in the digestive system and then circulates throughout the body. The microbes in the gut break down EGCG’s large polyphenol structures into smaller, active compounds, which can boost its effects throughout the body. It is mainly metabolized in the liver and colon and undergoes enterohepatic recirculation, allowing some of it to be reabsorbed. It is primarily eliminated via bile and urine, with only small traces of the original compound found in urine after ingestion. Several factors, such as fasting, how the compound is stored, levels of blood albumin and the intake of vitamin C, can influence how much EGCG enters the bloodstream and how available it is to the body. Conversely, bioavailability can decrease due to factors like oxidation, sulfation, glucuronidation, digestive breakdown and interactions with minerals like calcium, magnesium and trace metals. Genetic differences, such as variations in the COMT gene, can also impact how EGCG is processed. Importantly, when EGCG is taken with food, its absorption and availability (Cmax and AUC) are significantly reduced. This is partly because free EGCG is chemically unstable and poorly absorbed due to its strong antioxidant activity and complex chemical structure, which includes eight free hydroxyl groups. These properties make it prone to oxidation, gut metabolism and poor intestinal absorption [8,9]. Ullmann et al. (2003) assessed the safety, tolerability and pharmacokinetic properties of a single dose of EGCG, ranging from 50 mg to 1600 mg. They found that plasma concentrations of EGCG greater than 1 μM were only reached at doses above one gram (with a 1600 mg dose producing a Cmax of 3392 ng/mL, ranging from 130 to 3392 ng/mL). Peak concentrations were observed between 1.3 and 2.2 h after administration. The plasma kinetics of both free EGCG and total EGCG (which includes free EGCG and its conjugated metabolites) were tracked for 26 h following administration. Generally, doses of purified EGCG up to 1600 mg were well tolerated [18]. Chow et al. (2005) studied the safety and plasma kinetics of multiple doses of purified EGCG and the decaffeinated green tea extract, polyphenon E [10]. This research involved once-daily and twice-daily dosing regimens of EGCG and polyphenon E over four weeks. Similarly to the previous study, EGCG doses of 400 and 800 mg resulted in peak serum concentrations of both free and total EGCG in the high nanomolar range. However, chronic administration of 800 mg EGCG showed an increase in its bioavailability. Daily administration of EGCG led to only mild gastrointestinal side effects [13,19].

## 5. Application in Medicine

**Figure 2:**
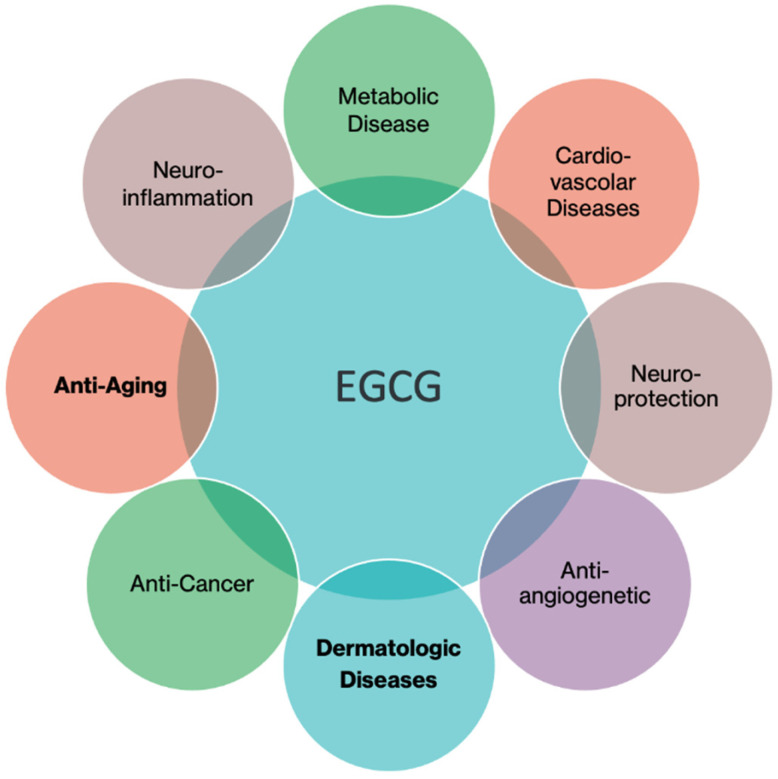
Multifaceted role of EGCG in anti-tumor activity, prevention of metabolic disorders, cardiovascular diseases, neuroprotection, prevention of neuroinflammation, anti-aging.

Studies report that the main catechin, the EGCG, is the most prevalent and biologically active molecule in green tea, playing a pivotal part in its health benefits, in particular: anti-tumor activity (against adrenal, bladder, breast, cervical, colorectal, esophageal, gastric, liver, lung, oral, ovarian, pancreatic, prostate and skin cancer), weight loss (obesity), prevention of metabolic disorders, cardiovascular diseases (heart failure, stroke, etc.), neuroprotection, prevention of neuroinflammation, anti-aging, etc. (Figure 2).

### 5.1. Anti-Tumor Activity

Epigallocatechin gallate (EGCG) has shown promising anti-cancer activity in both in vitro and in vivo studies across a wide range of tumor types, including adrenal, bladder, breast, cervical, colorectal, esophageal, gastric, liver, lung, oral, ovarian, pancreatic, prostate, and skin cancers. Most laboratory experiments used concentrations of EGCG ranging from 5 to 200 μM, demonstrating its ability to inhibit cancer cell proliferation, migration, adhesion, invasion, and metastasis, as well as suppress tumor-related angiogenesis. Beyond these general effects, EGCG is known to regulate molecular signaling pathways involved in cancer development. It suppresses pro-inflammatory responses by inhibiting phosphorylation of p38 and JNK kinases and by blocking NF-κB translocation to the nucleus. These actions reduce the expression of inflammation-related genes [20]. Additionally, EGCG plays a role in the elimination of misfolded proteins and cellular waste, which accumulate with aging and contribute to oncogenesis. EGCG also promotes apoptosis and enhances the immune system’s response to tumor cells, further strengthening its anti-tumor effects [21,22]. Specific attention has been given to EGCG’s role in breast cancer. It interferes with critical signaling cascades, such as PI3K/Akt and MAPK, and downregulates the Wnt pathway and miR-25, leading to growth arrest and apoptosis in cancer cells [23]. Researchers have developed several EGCG derivatives to improve stability and bioavailability, many of which have shown enhanced water solubility and stronger biological activity in experimental models. These modifications have resulted in derivatives capable of more effectively inhibiting proteasomal function, inducing apoptosis and reducing tumor growth in breast cancer models without harming healthy cells [24,25]. Lung cancer studies have also highlighted EGCG’s therapeutic potential, particularly in non-small cell lung cancer (NSCLC), the most common subtype. EGCG modulates multiple intracellular targets, such as ERK, VEGF, NF-κB, and AMPK, which are involved in cell proliferation and survival. When used in combination with traditional chemotherapy agents like cisplatin or paclitaxel, EGCG enhances therapeutic efficacy while potentially reducing drug dosage and side effects. Derivatives of EGCG have shown synergistic effects, particularly when targeting tumors with known genetic mutations such as those in the EGFR pathway. These derivatives often work by sensitizing tumor cells to chemotherapy through inhibition of repair enzymes and pro-survival pathways [26].

In endometriosis and other hormone-sensitive conditions like uterine fibroids, EGCG and its analogs have demonstrated strong anti-angiogenic properties without affecting reproductive tissue. One peracetylated derivative showed significant suppression of blood vessel formation in ectopic endometrial tissue in mouse models, pointing to its therapeutic potential beyond oncology [7]. The development of EGCG derivatives continues to focus on improving pharmacokinetic properties, targeting specific cellular pathways and minimizing systemic toxicity. These findings underscore the potential of EGCG and its synthetic analogs, not only as supportive agents in cancer therapy but also as lead compounds for the development of novel anti-cancer drugs (Table 1).

### 5.2. Weight Loss and Diabetes Mellitus

EGCG plays a significant role in weight loss by influencing several metabolic pathways that regulate fat accumulation, energy expenditure and appetite control. One of its key mechanisms is the activation of AMPK, an energy-sensing enzyme that promotes fat oxidation and reduces the synthesis of new fat in the liver and adipose tissue. By enhancing the breakdown of stored fats and simultaneously limiting their formation, EGCG helps shift the body toward a more favorable metabolic state for weight reduction. In a study conducted over 20 weeks, Li et al. (2018) gave mice either 50 mg/kg or 100 mg/kg of EGCG daily while being fed a high-fat diet. The treatment with EGCG led to a notable reduction in obesity, including a significant decrease in the weight of epididymal fat tissue. It also influenced key blood lipid parameters, lowering levels of triglycerides, total cholesterol, and both LDL and HDL cholesterol. Additionally, EGCG promoted the elimination of free fatty acids through feces. At the molecular level, analysis of gene expression revealed that EGCG suppressed the activity of genes responsible for the production of new fatty acids, such as acc1, fas, scd1, c/ebpβ, pparγ and srebp1. At the same time, it stimulated genes linked to fat breakdown, particularly hsl and those involved in fat oxidation within white adipose tissue. These changes were observed in both the high-fat diet group and the groups receiving EGCG, suggesting a regulatory effect of EGCG on lipid metabolism [27]. Another promising target for obesity intervention is the enzyme catechol-O-methyltransferase (COMT), which deactivates norepinephrine (NE) by methylation. Green tea, specifically EGCG, has been found to inhibit COMT, thereby prolonging NE’s activity and enhancing thermogenesis. This results in greater calorie expenditure, promoting weight reduction [28]. Researchers used a standardized green tea extract known as AR25 (EXOLISE^®^), made from unfermented *Camellia sinensis* leaves. Each 375 mg capsule contained 25% catechins (around 70% of which was EGCG) and 5–10% caffeine. In lab studies on rats, researchers tested the extract’s effect on brown adipose tissue (BAT), known for its role in heat generation. Results showed that the extract significantly increased oxygen consumption (MO_2_) in BAT, more than caffeine alone, especially when sympathetic nerve activity was stimulated with ephedrine. In rats with chemically damaged sympathetic nerves, the thermogenic effect was greatly reduced, showing that EGCG relies on the presence of NE. In further experiments, EGCG alone only mildly boosted BAT thermogenesis, but in combination with caffeine and ephedrine, the effect was synergistic, leading to a 2- to 7-fold increase in MO_2_. These findings suggest that EGCG does not cause more NE to be released, but prolongs its action, while caffeine enhances the NE signal by preventing cAMP breakdown [28,29]. A 3-month open-label trial in France tested the AR25 extract (80% ethanolic dry extract standardized at 25% catechins standardized to EGCG equivalents) on 70 moderately obese adults. Participants took two capsules (containing 375 mg each of green tea extract AR25) twice daily. By the end of the study, there was an average of 4.6% reduction in body weight and 4.5% reduction in waist circumference, with no adverse effects on blood pressure or heart rate. Although the study lacked a control group, the consistent weight and waist reduction across participants supports the potential of green tea extract as a natural aid for weight loss [30]. This body of research supports the idea that EGCG and caffeine in green tea work together through different biochemical pathways to enhance thermogenesis and potentially aid in weight management. Moreover, epigallocatechin gallate (EGCG) plays a potential adjunctive role in the prevention and management of diabetes, where it exerts antihyperglycemic effects by improving insulin sensitivity, reducing fasting blood glucose and modestly lowering HbA1c and HOMA-IR, shown by meta-analyses of randomized controlled trials. However, these effects are generally modest and not considered clinically meaningful as a stand-alone intervention for glycemic control in most populations [31]. Mechanistically, EGCG acts via several pathways: it enhances insulin secretion and beta cell function, reduces insulin resistance and protects pancreatic beta cells from high-glucose-induced mitochondrial apoptosis by modulating DRP1-mediated pathways [32,33]. EGCG also inhibits hepatic glucose production by downregulating PKA signaling and FoxO1, suppresses gluconeogenesis and glycogenolysis, and activates AMPK in hepatocytes [34]. Additionally, EGCG inhibits digestive enzymes such as α-amylase and α-glucosidase, thereby reducing postprandial glucose spikes [35,36]. These mechanisms share similarities with EGCG’s actions in cardiovascular disease, such as antioxidant and anti-inflammatory effects, improvement of endothelial function, and modulation of metabolic pathways [37,38,39]. However, in diabetes, the emphasis is on direct effects on glucose metabolism, insulin signaling and beta cell preservation, whereas in cardiovascular disease, the focus is more on vascular protection, lipid metabolism and anti-atherosclerotic actions.

### 5.3. Cardiovascular Disease

Over the past few decades, research has uncovered multiple mechanisms through which EGCG may exert cardioprotective effects, including anti-inflammatory, antioxidant, lipid-lowering and vasodilatory actions. Tadano et al. (2010) examined the direct effects of green tea catechins (GTCs) on heart muscle function. Using techniques such as quartz crystal microbalance (QCM) and nuclear magnetic resonance (NMR), they found that EGCG and ECG, but not their stereoisomers (−)-catechin-3-gallate and (−)-gallocatechin-3-gallate, reduced calcium sensitivity in cardiac myofilaments, likely through interaction with cardiac troponin C. In a mouse model of hypertrophic cardiomyopathy, EGCG was able to restore cardiac output. These findings suggest that EGCG and ECG may counteract the excessive calcium sensitivity of cardiac myofilaments observed in hypertrophic cardiomyopathy [40,41]. One of the central mechanisms by which EGCG supports cardiovascular health is through its potent antioxidant activity. Oxidative stress plays a critical role in the development of atherosclerosis and other cardiovascular conditions, largely by promoting the oxidation of low-density lipoprotein (LDL) and contributing to endothelial dysfunction. EGCG can directly scavenge reactive oxygen species (ROS) and enhance the body’s endogenous antioxidant defense systems, such as superoxide dismutase and glutathione peroxidase. This reduction in oxidative stress not only protects vascular cells but also slows the progression of atherosclerotic plaque formation. Momose et al. (2016) investigated the cholesterol-lowering potential of EGCG by administering doses ranging from 107 to 856 mg per day over a period of 4 to 14 weeks. Their findings indicated that EGCG intake significantly reduced LDL cholesterol levels. Additional evidence suggests that catechins, including EGCG, help regulate cholesterol by inhibiting its synthesis and enhancing its excretion, which contributes to more stable cholesterol levels and reduced lipid accumulation in the body [42,43]. In addition to combating oxidative damage, EGCG exerts significant anti-inflammatory effects. Chronic inflammation is a key driver of cardiovascular pathology, contributing to endothelial activation, leukocyte adhesion and plaque instability. EGCG has been shown to suppress pro-inflammatory cytokines like tumor necrosis factor-alpha (TNF-α) and interleukin-6 (IL-6), while also inhibiting critical inflammatory signaling pathways, including NF-κB and MAPK. Wang et al. (2014) discovered that EGCG can interfere with the inflammatory process in human vascular endothelial cells by blocking the NF-κB signaling pathway, which is activated through the 67 kDa laminin receptor (67LR). This blockage leads to a reduction in the production of MCP-1, a key inflammatory molecule involved in vascular inflammation. The MAPK signaling pathway is another important regulatory system that controls processes such as cell growth, differentiation, transformation, and programmed cell death. Within this pathway, three major kinases—ERK1/2, JNK, and p38 MAPK—play critical roles in determining whether endothelial cells are protected or damaged. ERK1/2 is usually triggered by phosphorylation of its upstream molecule MEK, influencing cell growth and differentiation. In contrast, p38 MAPK and JNK are typically activated in response to cellular stress and are involved in mediating inflammation and apoptosis. These stress-activated kinases can also interact with the NF-κB pathway, forming a feedback loop that intensifies inflammatory gene expression and contributes to the progression of atherosclerosis [44]. EGCG has been shown to bind to 67LR and suppress the activation of all three MAPK pathway components, ERK1/2, p38 and JNK, thereby reducing inflammation and cell stress responses in endothelial cells. Supporting this, Yang and his team demonstrated that EGCG, at concentrations between 5 and 25 µmol/L, could prevent the activation of p38 MAPK and JNK1/2 in HUVECs exposed to angiotensin II, a compound known to induce endothelial dysfunction. Similarly, Chae and colleagues found that EGCG, at doses ranging from 10 to 50 µmol/L, inhibited the phosphorylation of both p38 MAPK and ERK, thereby reducing inflammation and preventing adhesion of inflammatory cells to the endothelium [44]. Moreover, EGCG exhibits antithrombotic and anti-platelet properties, which are essential in preventing the formation of blood clots that can lead to myocardial infarction or stroke. It reduces platelet aggregation and downregulates the expression of adhesion molecules, thereby supporting normal blood flow and reducing thrombotic risk. EGCG may also enhance fibrinolytic activity, further contributing to cardiovascular protection.

Lill et al. (2003) studied how six different catechins (C, EC, EGC, CG, ECG and EGCG) affect platelet activity. They found that, among these compounds, only EGCG was effective in preventing platelet aggregation triggered by thrombin in a laboratory setting [45]. Platelets, like many other cells in the body, metabolize arachidonic acid (AA), which leads to the formation of prostaglandin endoperoxides PGG2 and PGH2 through the action of cyclooxygenase-1 (COX-1). PGH2 is then converted into thromboxane A2 via thromboxane synthase, a process central to platelet activation and aggregation. Interestingly, EGCG has been shown to inhibit COX-1 activity even more effectively than aspirin. In experimental models where platelet aggregation was induced by collagen, EGCG reduced thromboxane A2 production by lowering COX-1 activity, demonstrating a strong anti-aggregatory effect [46]. Beyond COX-1 inhibition, EGCG influences platelet function through multiple mechanisms. It interferes with collagen-triggered activation of phospholipase C gamma 2, disrupts protein tyrosine phosphorylation (a key step in platelet signaling), and enhances the activity of calcium ATPase, which helps regulate intracellular calcium levels. These actions collectively reduce platelet aggregation and help prevent thrombus formation, highlighting EGCG’s potential as a natural antithrombotic agent [47].

### 5.4. Neuroprotection

Promising results have been seen in Alzheimer’s Disease and Parkinson’s Disease, the two most prevalent types of neurodegenerative disorders. In the case of Alzheimer’s disease (AD), key pathological features include the build-up of Amyloid Beta (Aβ) peptides, problems with mitochondrial function, inflammation-related synaptic damage, increased levels of reactive oxygen species (ROS) and impaired blood flow in the brain. The dual role of epigallocatechin-3-gallate (EGCG) consists of reducing brain inflammation and protecting neurons. Recognized as a nutraceutical, EGCG has shown encouraging potential in slowing the progression of AD due to its antioxidant, anti-inflammatory, and anti-aging properties [48]. A study conducted by Mori et al. (2019) showed that EGCG helped reverse learning and memory impairments in a transgenic mouse model of Alzheimer’s disease (APP/PS1). Treatment with EGCG reduced the accumulation of amyloid plaques in the brain and encouraged the processing of amyloid precursor protein (APP) through non-amyloidogenic pathways. It also alleviated brain inflammation, reduced gliosis (an abnormal increase in glial cells) and lessened oxidative stress. Moreover, EGCG boosted the activity of α- and β-secretases, decreased neuroinflammation, and supported the restoration of oxidative balance in the brain. The study also reported that EGCG elevated levels of enzymes such as ADAM10 and furin, which are associated with non-amyloidogenic APP processing and inhibition of BACE1, a key enzyme in amyloid plaque development. In a separate study by Andrade and colleagues, EGCG was shown to have anti-amyloid properties by promoting the breakdown of Aβ fibrils in neuronal membranes, likely due to its moderate binding to lipid membranes. Additionally, EGCG was found to interact with α-synuclein, helping to block the formation of amyloid fibrils involved in Parkinson’s disease progression [49]. In another study, So-Ra Kim et al. (2022) showed a possible role of ECG in reducing Hypoxia-Induced Inflammation in Microglia via NF-κB Suppression and Nrf-2/HO-1 activation. Hypoxia is a key contributor to several neurodegenerative and neurological diseases, including stroke, Alzheimer’s disease, Parkinson’s disease and epilepsy. Under hypoxic conditions, microglia, immune cells in the central nervous system, can become activated into a pro-inflammatory M1 state, releasing cytokines such as TNF-α, IL-1β and IL-6, and producing reactive oxygen species (ROS), which contribute to neuronal damage. Conversely, M2 microglia promote recovery by secreting anti-inflammatory factors like IL-10 and TGF-β, controlling inflammation under hypoxic conditions and, consequently, preventing and treating neuroinflammatory diseases [50,51]. This study investigates the anti-inflammatory mechanisms of EGCG in microglial cells exposed to CoCl_2_, which mimics hypoxia. Findings show that CoCl_2_ treatment increases pro-inflammatory mediators (IL-6, iNOS, COX-2), activates NF-κB p65, elevates HIF-1α (Hypoxia-inducible factor-1α) expression and enhances ROS production, all of which contribute to inflammation and cell death. EGCG pre-treatment significantly reduces these effects. In particular, it inhibits NF-κB activation and its downstream inflammatory gene expression, suppresses HIF-1α expression, reduces ROS production, activates the Nrf2/HO-1 antioxidant pathway, which is normally suppressed under hypoxia, enhancing cellular defense against oxidative stress and protects against hypoxia-induced apoptosis, as shown by reduced caspase-3 and PARP cleavage. These results suggest that EGCG exerts anti-inflammatory, antioxidant and anti-apoptotic effects by modulating multiple pathways, including NF-κB, HIF-1α/ROS and Nrf2/HO-1. Its ability to cross the blood–brain barrier and cause fewer side effects than traditional anti-inflammatory drugs makes EGCG a promising neuroprotective agent [50,52]. Moreover, EGCG has been shown in animal studies to reduce stress and anxiety and to promote calming and sleep-inducing effects in a dose-dependent manner. It interacts with the brain’s GABA_A_ receptors, which are key to calming neural activity. Specifically, EGCG enhances the effects of diazepam (a common anti-anxiety medication) on these receptors at low doses (around 0.1 μM), without affecting GABA function on its own. However, at higher doses (15 μM), EGCG can actually inhibit GABA’s action, showing a complex, concentration-dependent effect. There is also evidence that EGCG and other flavonoids can interact synergistically with compounds affecting GABA and glycine receptors, potentially influencing mood and behavior. In human studies, drinking a cup of GABA-enriched oolong tea was shown to reduce stress. Chemical analysis showed that this GABA tea contained eight times more GABA, but ten times less EGCG and slightly more caffeine than standard tea. This reflects typical tea preparation methods (i.e., steeping in hot water), reflecting realistic consumption levels. Other research using more intense extraction methods reported smaller changes in EGCG content. The decrease in EGCG in GABA-enriched tea may be important because EGCG plays a significant role in modulating GABA receptors. This suggests that regular oolong tea’s calming effects may rely more on EGCG, while the benefits of GABA-enriched tea are likely due to its high GABA content, not EGCG [53].

## 6. Application in Dermatology

EGCG has numerous applications in dermatology. Several studies have reported its beneficial effects in the treatment of external genital warts and perianal warts, acne, psoriasiform and interface dermatitis and androgenetic alopecia. New perspectives are also emerging in aesthetic medicine, in reducing UV-induced oxidative stress, modulating autophagy and inflammatory responses, promoting skin health, hair and nail improvement and rejuvenation [54,55,56].

### 6.1. Treatment of Genital and Perianal Warts

**Figure 3:**
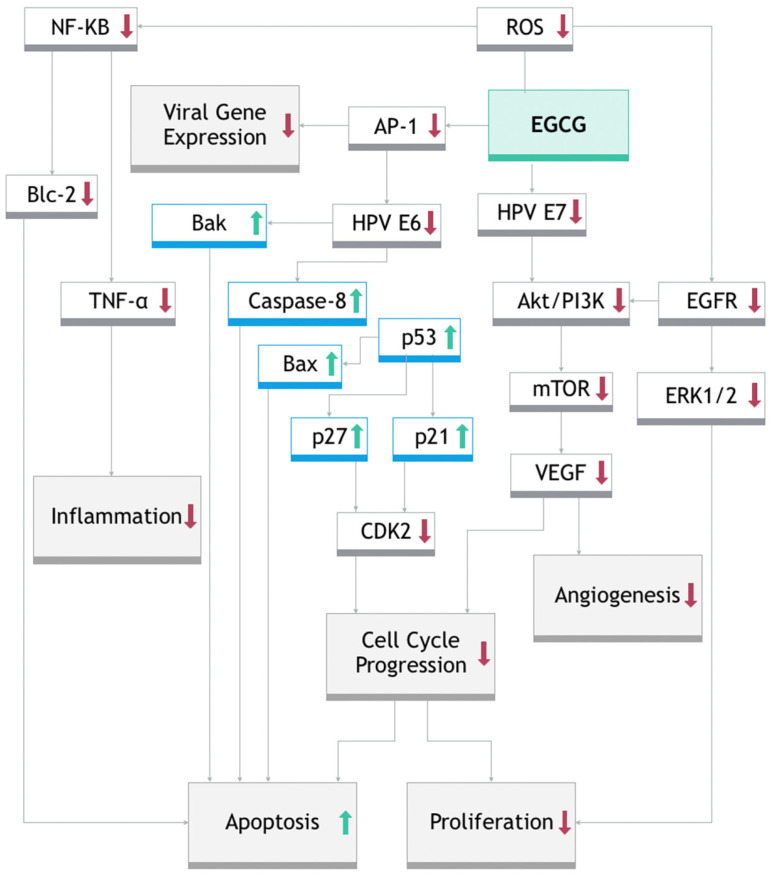
Mechanism of action of EGCG in cell cycle block and suppression of cell growth. (Green upward arrows indicate an upregulation, whereas red downarrows indicate a downregulation).

EGCG showed good results in the treatment of external genital and perianal warts. Indeed, Polyphenon^®^E is a standardized green tea catechin (GTC) formulation, approved by the U.S. Food and Drug Administration (FDA) in 2006, as a treatment for genital warts, under the brand name Veregen^®^ in immunocompetent patients aged 18 and older. Its posology is three times a day until the warts are completely cleared or for a maximum duration of 16 weeks [54]. EGCG can halt the cell cycle, suppressing cell growth, through 2 mechanisms of action: triggering programmed cell death (apoptosis) and inhibiting the formation of new blood vessels (angiogenesis). This is achieved by downregulating several key molecules, including the HPV oncogenes E6 and E7, the epidermal growth factor receptor (EGFR), the AKT/PI3K signaling pathway, mTOR, and ERK1/2. When EGCG suppresses E6, it leads to an increase in tumor-suppressor proteins (p53, p21, and p27), causing a reduction in CDK2, a cyclin-dependent kinase, ultimately resulting in cell cycle arrest. Additionally, EGCG-induced upregulation of p53 can activate another pro-apoptotic protein, Bak, further promoting apoptosis. EGCG may exert its effects largely through its antioxidant activity (see Figure 2), since reactive oxygen species (ROS) can activate nuclear factor-κB (NF-κB), which drives the expression of inflammatory cytokines like TNF-α and IL-1β, supports cancer cell growth and invasion, and increases the levels of the anti-apoptotic protein Bcl-2. EGCG, by scavenging ROS, may reduce Bcl-2 expression and promote apoptosis in HPV-infected skin cells (Figure 3) [55,57].

### 6.2. Treatment of Acne Vulgaris

EGCG shows significant promise as a therapeutic agent in acne due to its multifaceted effects on the key pathological processes involved in the disease. Acne is characterized by excessive and altered sebum production (hyperseborrhea and dysseborrhea), follicular hyperkeratinization, inflammation, and proliferation of *Cutibacterium acnes*. These processes are primarily driven by hormonal influences, especially androgens, insulin and IGF-1, which activate signaling pathways such as PI3K/Akt/mTOR and suppress FoxO1, a negative regulator of lipid synthesis and inflammation. EGCG plays a central role in counteracting these mechanisms. It inhibits IGF-1-induced lipogenesis in sebocytes by suppressing mTOR and its downstream target, S6 ribosomal protein, both of which are vital components of the Akt pathway. Additionally, EGCG activates AMPK, which in turn inhibits SREBP-1, a transcription factor essential for lipid synthesis. This leads to a reduction in sebum production, targeting the root of sebocyte-driven acne pathology [57]. Beyond regulating lipid metabolism, EGCG exerts anti-inflammatory effects by suppressing pro-inflammatory cytokines such as IL-1α and IL-6 in sebocytes. This is particularly relevant since IGF-1 not only stimulates sebum production but also triggers inflammatory responses. Furthermore, EGCG may also benefit keratinocytes by reducing IL-1 levels, helping to prevent hyperkeratinization and comedone formation [58,59]. Thus, EGCG influences both the metabolic and inflammatory pathways central to acne development. Its ability to modulate mTORC1 and activate AMPK positions it as a potentially effective and natural alternative or adjunctive treatment for acne, especially in cases where traditional therapies may not be well tolerated or desired [58,60]. Mohamed L. Elsaie et al. evaluated the efficacy and safety of a topical green tea lotion in individuals with mild to severe acne. Over a six-week period, participants applied the formulation twice daily. Results indicated a statistically significant reduction in acne severity, with the severity index (SI) decreasing by 39.02% (*p* < 0.0001). Adverse events were minimal and transient, limited to mild pruritus and a temporary stinging sensation in a small subset of participants [61]. The study further supports the mechanistic rationale for the use of green tea in acne management. EGCG has been shown to downregulate matrix metalloproteinases (MMP-2 and MMP-9), which are elevated in acne lesions and contribute to dermal degradation and scarring. Additionally, green tea polyphenols exhibit potent antioxidant activity, counteracting the deleterious effects of reactive oxygen species (ROS) that contribute to lipid peroxidation, inflammation, and comedogenesis. These antioxidative properties parallel those of several conventional acne therapies such as tetracycline and erythromycin, though green tea offers a more favorable safety profile [62,63].

### 6.3. Treatment of Hair Disorders

Epigallocatechin gallate (EGCG) has gained significant attention for its potential role in promoting hair growth, improving overall hair health and counteracting androgenetic alopecia. One of its key mechanisms involves the inhibition of 5α-reductase. By reducing DHT levels, EGCG may help slow or prevent hair follicle miniaturization. Additionally, EGCG supports the health and proliferation of dermal papilla cells (DPCs), essential for regulating hair growth and the hair cycle [64]. Studies show that EGCG activates the ERK and Akt signaling pathways in these cells, promoting cell survival and proliferation while reducing apoptosis. This activity may help extend the anagen (growth) phase of the hair cycle, leading to longer and thicker hair. EGCG also exhibits anti-inflammatory and antioxidant properties, which help protect hair follicles from oxidative stress and inflammation—two factors commonly associated with hair loss. These combined effects make EGCG a promising natural compound for improving scalp health and supporting hair growth, both in preventive care and as part of therapeutic strategies for hair loss conditions [65]. In organ culture, isolated human hair follicles treated with EGCG exhibited significant elongation over a 10-day period. The extent of hair follicle growth was found to increase in a dose-dependent manner, with the highest concentration of EGCG (5 µM) resulting in over 180% elongation compared to vehicle-treated controls. These findings suggest a direct stimulatory effect of EGCG on the hair growth cycle, likely promoting or sustaining the anagen phase. Complementary to this, in vitro experiments using cultured human dermal papilla cells showed that EGCG enhanced cellular proliferation, as assessed using an MTT assay. The effect was again dose-dependent, with statistically significant increases observed at all tested concentrations. To explore the molecular mechanisms underlying these proliferative effects, the researchers analyzed the expression of key signaling proteins using Western blotting. They found that EGCG activated both the ERK and Akt signaling pathways, which are known to be involved in cell proliferation and survival. This activation was evidenced by increased levels of phosphorylated ERK and phosphorylated Akt in EGCG-treated cells. Furthermore, EGCG altered the expression of apoptosis-related proteins, increasing the expression of the anti-apoptotic protein Bcl-2, while simultaneously decreasing the pro-apoptotic protein Bax. These molecular changes indicate that EGCG not only stimulates DPC proliferation but also supports cell survival by suppressing apoptosis [66]. To confirm whether these cellular and molecular effects could be replicated in a living system, the study included a small in vivo trial in which a 10% EGCG solution in ethanol was topically applied to the occipital scalp of three healthy male volunteers over four consecutive days. Subsequent tissue analysis from the treated scalp regions revealed similar molecular changes to those observed in vitro, including upregulated phosphorylated ERK and Akt, increased Bcl-2 expression, and reduced Bax expression. These results confirmed that EGCG is capable of penetrating the scalp and activating the same pro-growth and anti-apoptotic pathways in vivo [66,67]. Moreover, its strong antioxidant and anti-inflammatory properties also help to protect the nail matrix from damage caused by oxidative stress and inflammation. This is particularly important, as environmental factors, aging, and nutritional deficiencies can all weaken nails and slow their growth. EGCG may also support healthy keratin production, the key structural protein that makes up nails, skin and hair. By promoting keratinocyte function and reducing inflammatory mediators, EGCG could contribute to stronger, smoother, and less brittle nails. Moreover, thanks to its antimicrobial properties, EGCG may help protect nails from fungal infections, which are a common cause of nail discoloration, thickening, and breakage. While research on EGCG’s specific effects on nails is still emerging, its known biological actions suggest it may be a valuable natural ingredient in nail-strengthening treatments and supplements aimed at improving overall nail quality and resilience [65].

## 7. Association with Other Supplements

Numerous studies have shown that combining EGCG with other supplements enhances its effectiveness and further expands the potential applications of this versatile molecule. Reported benefits include stress reduction, improved sleep, decreased pain and discomfort, along with many other positive effects [68,69].

### 7.1. Association with B-Group Vitamins

It has been observed that combining EGCG with B vitamins—especially when paired with other compounds naturally found in green tea, such as L-theanine—effectively reduced stress levels by days 14 and 28 in individuals experiencing chronic stress but otherwise in good health. Additionally, this combination appears to lessen the perception of pain, highlighting its potential usefulness for chronic pain patients, a condition often accompanied by stress and sleep disturbances. These promising results warrant further investigation in future research [68].

### 7.2. Association with Vitamin D

The use of EGCG is paving the way for innovative approaches in the treatment of uterine myomas. Research indicates that EGCG, especially when combined with Vitamin D, may be an effective option for managing both the growth of myomas and their related symptoms. Several studies highlight the synergistic effect of this combination as a promising alternative therapy. In one study, total myoma volume was reduced by 34.7% in the treatment group, while it increased by 6.9% in the control group. Women receiving a combination of EGCG, Vitamin D, and Vitamin B6 also reported improved quality of life alongside the reduction in myoma size. Importantly, this non-surgical approach may help women preserve fertility and lower the risk of further gynecological issues [69].

### 7.3. Association with β-Cryptoxanthin

As previously mentioned, the combination of EGCG—a natural compound in green tea—and β-cryptoxanthin—present in citrus fruits—has been shown to act synergistically in combating obesity. When taken together over a four-week period, compounds led to a significant decrease in body weight and a marked reduction in both the volume and weight of white adipose tissue [70].

### 7.4. Association with Caffeine

Studies have shown that administering tea or EGCG to animals can suppress chemically induced cancer development in various animal models. This effect appears to be enhanced when EGCG is combined with caffeine. While caffeine alone has demonstrated cancer-inhibiting properties in several models, in some cases it has been found to promote carcinogenesis. The exact reasons why caffeine produces protective effects in some models but has the opposite effect in others remain unclear [71,72].

## 8. Role of ECG in Skin Anti-Aging

EGCG plays a significant role in anti-aging due to its strong antioxidant, anti-inflammatory, and cell-protective properties. One of the primary mechanisms through which EGCG supports healthy aging is by neutralizing free radicals, unstable molecules that contribute to cellular damage, oxidative stress, and accelerated aging of the skin (wrinkles, hyperpigmentation and loss of elasticity). It also supports the production of collagen and elastin, essential proteins for maintaining firm, youthful skin, while also inhibiting enzymes that degrade these structural components over time [72,73]. Moreover, EGCG works by modulating cellular processes such as autophagy, a critical biological mechanism that facilitates the degradation and recycling of damaged proteins, lipid droplets, and organelles through lysosomal pathways. This process is essential for maintaining cellular homeostasis, especially under stress conditions, and plays a protective role in a variety of diseases, including cancer and metabolic disorders [15,74]. In skin cells, the activation of autophagy can help remove aged or dysfunctional cellular components, thus supporting tissue renewal and repair. EGCG has been found to effectively induce this process by influencing key regulatory pathways, including the inhibition of the mammalian target of rapamycin (mTOR) and the activation of AMP-activated protein kinase (AMPK) and mitogen-activated protein kinase (MAPK). Moreover, EGCG contributes to skin rejuvenation by reducing the activity of negative regulators such as GADD34, which are involved in promoting apoptosis (programmed cell death), helping to preserve the viability and function of skin cells. Together, these actions suggest that EGCG not only protects skin cells from age-related damage but also enhances their capacity for renewal and repair [15]. Topically, EGCG is used in various skincare formulations to reduce fine lines, improve skin texture, and even out skin tone [65,75].

EGCG has also shown strong protective effects against UV-induced skin damage and photocarcinogenesis in animal studies. In recent research, even a single exposure of human skin to ultraviolet (UV) radiation was found to trigger the production of reactive oxygen species (ROS), with increased levels of molecules such as hydrogen peroxide (H_2_O_2_) and nitric oxide (NO) in both the epidermis and dermis. However, when EGCG was applied topically before UV exposure, it significantly lowered the levels of these oxidative stress markers in both skin layers, demonstrating its effective antioxidant protection [73]. In various experiments, green tea was either applied directly to the skin or included in the diet of mice prior to UV exposure. Both methods led to a noticeable decrease in tumor development compared to untreated controls. In a separate study, rats treated with EGCG showed a marked reduction in sunburn cell formation following UV radiation [17,75]. EGCG has also shown promising effects in skin-brightening. When modified through glucosylation to form EGCG-G1, its skin absorption and potential bioavailability improve, possibly through interaction with the GLUT1 transporter. Its ability to lighten skin is linked to the suppression of melanin-related genes and the stimulation of beneficial bacteria like Lactobacillus, which generate compounds with skin-brightening effects. EGCG also reduces the expression of MITF and TYR, key proteins in melanin production, and interferes with the MC1R signaling pathway, slowing the development of melanosomes [76]. These findings suggest that EGCG holds potential as an ingredient in topical depigmenting treatments, opening new possibilities for managing pigmentation disorders and enhancing skin tone.

## 9. Conclusions

Overall, EGCG is increasingly recognized as a valuable natural compound for both topical and oral applications, contributing not only to longevity but also to healthier, more resilient, and visibly rejuvenated skin over time. Daily oral intake has been shown to provide a wide range of benefits—supporting general health, enhancing physical well-being, and improving appearance—while also helping to slow down various aspects of the aging process. Supplementation of up to 1600 mg per day is generally well tolerated in healthy individuals, with no adverse effects on liver function. For these reasons, we aimed to highlight its benefits, with the hope that what are now considered traditional Eastern practices—such as the tea ritual and tea breaks, particularly popular in Japan and China—will increasingly become part of daily wellness routines in the Western world as well. In this context, EGCG could serve as a fundamental component of holistic health strategies, while topical formulations continue to support the treatment of various skin concerns.

## Figures and Tables

**Table 1:** Main EGCG anti-tumoral implications and their mechanism of action (Upward arrows indicate an upregulation, whereas downarrows indicate a downregulation).

Type of Tumor	Mechanism of Action of EGCG		
Adrenal	↓ anti-apoptotic protein (Bcl-2, Bcl-xl, xIAP and cIAP	↑ pro-apoptotic factors (Bad, Bax, Fas/CD95)	Activation of caspases -3, -7, -8 and -9
Bladder	Inhibition of PI3K/AKT pathway and upregulation of PTEN	Caspase activation and modulation of BCL-2 family proteins	Suppression of NF-κB and MMP-9
Breast	↓ EGFR/Src, PI3K/Akt and STAT3 pathways	↓ Wnt pathway and miR-25	↑ epithelial markers (E-cadherin)
Colorectal	↓ cyclins (Cyclin D1, Cyclin E) and CDKs (CDK2, CDK4, CDK6)	Inhibition of Wnt/β-catenin pathway	↓ VEGF expression and suppression of HIF-1
Gastric	↓ cyclins (Cyclin D1, Cyclin E) and CDKs (CDK2, CDK4 and CDK6)	Mitochondrial (intrinsic) pathway activation: ↑ Bax, ↓ Bcl-2	Inhibition of PI3K/Akt/mTOR pathway
Lung	Inhibits EGFR phosphorylation	↓ cyclins (Cyclin D1, Cyclin E), and CDKs (CDK2, CDK4, CDK6)	Mitochondrial (intrinsic) pathway activation: ↑ Bax, ↓ Bcl-2
Ovarian	↓ VEGF expression and suppression of HIF-1α	↓ metalloproteinases MMP-2, MMP-9	Inhibition of Wnt/β-catenin pathway
